# Measurement of matrix metalloproteinase 9-mediated Collagen type III degradation fragment as a marker of skin fibrosis

**DOI:** 10.1186/1471-5945-11-6

**Published:** 2011-03-29

**Authors:** Efstathios Vassiliadis, Sanne Skovgård Veidal, Natasha Barascuk, Jhinuk Basu Mullick, Rikke Elgaard Clausen, Lise Larsen, Henrik Simonsen, Dorthe Vang Larsen, Anne-Christine Bay-Jensen, Toni Segovia-Silvestre, Diana Julie Leeming, Morten A Karsdal

**Affiliations:** 1 Assay Development, Nordic Bioscience, Herlev Hovedgade 207, DK-2730, Copenhagen, Denmark; 2 Dept. of Endocrinology, University of Southern Denmark, Kløvervænget 6, DK-5000, Odense, Denmark; 3 Faculty of Science, Institute of Biology, University of Copenhagen, Tagensvej 16, DK-2200, Copenhagen Denmark

## Abstract

**Background:**

The current study utilized a Bleomycin-induced model of skin fibrosis to investigate the neo-epitope CO3-610 (KNGETGPQGP), a fragment of collagen III released during matrix metalloproteinase-9 (MMP9) degradation of the protein, we have previously described as a novel biomarker for liver fibrosis. The aim was to investigate CO3-610 levels in another well characterised model of fibrosis, to better describe the biomarker in relation to additional fibrotic pathologies.

**Methods:**

Skin fibrosis was induced by daily injections of Bleomycin to a total of 52 female C3 H mice, while control mice (n = 28) were treated with phosphate buffered saline (PBS), for 2, 4, 6 or 8 weeks. Skin fibrosis was evaluated using Visiopharm software on Sirius-red stained skin sections. Urine ELISA assays and creatinine corrections were performed to measure CO3-610 levels.

**Results:**

CO3-610 levels were significantly higher in Bleomycin-treated vs. PBS-treated mice at each time point of termination. The mean increases were: 59.2%, P < 0.0008, at 2 weeks; 113.5%, P < 0.001, at 4 weeks; 136.8%, P < 0.0001 at 6 weeks; 157.2%, P < 0.0001 at 8 weeks). PBS-treated mice showed a non-significant increase in CO3-610 levels (mean increase for weeks 2-8 = 1.7%, P = 0.789) CO3-610 levels assayed in urine were statistically significantly correlated with Western blot analysis showing increased skin fibrosis (P < 0.0001, r = 0.65).

**Conclusion:**

Increased levels in mouse urine of the MMP-9 mediated collagen III degradation fragment CO3-610 were correlated with skin fibrosis progression, suggesting that CO3-610 may be a potential positive biomarker to study the pathogenesis of skin fibrosis in mice.

## Background

The extracellular matrix (ECM) is the major component of connective tissue. It consists mainly of proteoglycans, glycoprotein and collagens, all of which have important and unique roles in maintaining the physicochemical structure of tissue [1-3]. Fibrotic skin diseases share a number of common phenotypic manifestations, particularly the accumulation of collagen, indicating a disturbed balance in ECM remodelling (ECMR) [4]. Matrix metalloproteinases (MMPs) play a central role in the proteolytic degradation of collagens and other extracellular molecules, resulting in the generation of specific cleavage fragments of proteins which in turn produce new epitopes. These neo-epitopes may be used as disease markers in a specific organ or in a specific disease.

In fibrosis, which may be described as extensive scar formation, the central pathological change is uncontrolled ECM remodelling [5,6]. This remodelling leads to the accumulation of fibrous (scar) tissue and an overall increase in ECM density[7]. Histopathological examination of biopsies is the traditional gold standard for diagnosis and staging fibrosis and is of utmost significance when evaluating the effect of therapeutic intervention. Biopsy, however, has significant drawbacks. It is invasive and prone to sampling error due to variation in the length and size of the tissue specimen, which subsequently leads to low reproducibility and high intra-patient variation.

Non-invasive biomarkers of fibrogenesis are available for diagnosis and follow-up of fibrosis, but their accuracy is highly variable and in essence they are not tissue specific. Collagen type III and its N terminal propeptide (PIINP) have been shown to be potentially valuable markers of collagen turnover [8] with significance not only for liver fibrosis but potentially for fibrotic skin [9].

Several animal models of skin fibrosis have been developed including models utilising exogenous application of growth factors [10] and the chromosome 2 dominant mutation of tight skin (Tsk) [11,12]. However, the Bleomycin-induced fibrosis model established by Yamamoto [13-17] is the predominant model of skin fibrosis and was chosen for the current study. Bleomycin is an anti-tumor agent due to its ability to inhibit DNA synthesis. It causes single strand scission of DNA in vivo and in vitro at specific base sequences. Its ability to cleave double-stranded DNA requires the presence of iron and oxygen. It also cleaves RNA to a lesser degree and in a more highly selective fashion.

The aim of the current study was to investigate whether an ELISA sensitive for a MMP-9 mediated type III collagen degradation neoepitope, C03-106, would be useful as a marker of skin fibrosis.

## Methods

### Animals

80 female C3 H mice were housed at the animal research facilities at Nordic Bioscience, Beijing, China in standard type III H cages at 22°C ± 2°C with relative humidity 50 ± 20% with bedding and nest material. The animals were kept under conditions of a 12-hour light/dark cycle, ventilated with filtered non-recycled air. Their diet consisted of standard food pellets and MilliQ water for the entire test period. The study was approved by the Danish animal ethics council regulated by the Danish ministry of justice (Dyreforsøgstilsynet) (study approval number: 2008/561-1450).

### Study design

In 52 C3 H mice, skin fibrosis was induced by daily Bleomycin injections into their backs (10 μg, 7 days a week). Dosing volume was 100 μl with the Bleomycin concentration being 100 μg/mL. 28 mice were injected with PBS daily into their backs and served as controls. The animals were divided into 4 groups, each comprising 7 PBS- and 13 Bleomycin-treated mice: Group 1 was sacrificed after 2 weeks, Group 2 after 4 weeks, Group 3 after 6 weeks, and Group 4 was sacrificed after 8 weeks. On completion of each study period, and following 14 hours of fasting, the animals were asphyxiated by carbon dioxide and sacrificed by exsanguinations.

### Urine and serum sampling

Urine and serum samples were taken from animals fasted for at least 14 hours overnight. Samples were collected at the following time points. All animals: baseline (day 0); Group 1: Day 9, 16 (termination); Group 2: Day 9, 16, 23, 30 (termination); Group 3: Day 9, 16, 23, 30, 37, 44 (termination); Group 4: Day 9, 16, 23, 30, 37, 44, 51, 58 (termination). Blood samples were taken from the retro-orbital sinus of the mice under light CO2/O2 anesthesia at baseline and at termination. Blood was collected in plain tubes and left at room temperature for 30 minutes to clot, then centrifuged at 1500 g for 10 minutes. All clot-free liquid was transferred to a new Eppendorf tube and centrifuged at 1500 g for 10 minutes. Serum was then transferred to a clean Eppendorf. Urine and serum were stored at -80°C in labeled Eppendorf tubes.

### Tissue handling

Skin sections from the injected area were carefully dissected, weighed, fixed in 4% formaldehyde for a minimum of 24 hours, cut into slices and embedded in paraffin. An additional skin section from the injected area was excised and stored at -80°C for protein extractions. 5 μm slices were cut, mounted on glass slides and stained with a combination staining protocol for Sirius red and Alcian blue. Skin sections were examined histologically for accumulation of collagen and presence of proteoglycans.

### Immunohistochemistry

5 μm sections of tissue in paraffin were cut, left for 1 hour at 60°C, dried overnight at 37°C, and stored at 4°C for later use. As and when samples were required, they were firstly heated at 60°C for 1 hour to melt the paraffin. Sections were then deparaffinized in toluene and rinsed in 99% ethanol 2 times for 5 minutes each. Slides were left for 20 minutes at room temperature in a 1.05% hydrogen peroxide in 99.9% ethanol. Slides were rehydrated in a series of solutions containing from 96% to 70% ethanol and rinsed in MilliQ water. Skin sections were pretreated with citrate buffer (pH 6) in a microwave oven (2 × 5 min at 800 W) and left for 20 minutes at room temperature. Primary antibodies were diluted according to the manufacturer's (AbCam, Cambridge, UK) guidelines with 1% bovine serum albumin and left on slides for 30 minutes, after which they were washed in 0.1% Triton X-100 (Sigma Aldrich, T8787, St. Louis, Missouri, USA) (2 × 5 min). Secondary antibody (Polymer-HRP, SS Label, Cat No HK519-XAK, and BioGenex, California, USA) reactions were blocked with 5% mouse serum for 1 hour, followed by incubating the slides for 30 minutes. Slides were washed again in 0.1% Triton X-100 (2 × 5 min). After a final wash at room temperature in 0.1% Triton X-100 (2 × 5 min), the last step was to incubate the slide with AEC substrate for 10 minutes. Slides were then rinsed with MilliQ water, counter-stained with Mayer's hematoxylin, mounted in Kaiser and air-dried overnight under air hood. A system utilizing HistoMark (KPL, Cat No. 70-00-18 & 71-00-19, Gaithersburg, USA) was also used; however this increased the cross-reactivity with mouse immunoglobin. Aggrecan, Biglycan, Versican and Decorin antibodies used for immunohistochemistry were the same as those described for Western blot analysis in the relevant section below.

### Biotinylation of antibodies

1 ml of 1 mg/ml purified antibody in PBS buffer was transferred to a 4 ml mini-sorb tube, and 110 μl of 1 M NaCO_3_/NaHCO_3 _(pH 9.6) was added. 8 μl of 0.1 M biotinylation reagents consisting of 10 mg BxNHS dissolved in 220 μL DMSO was added and the mixture incubated at room temperature for 1 hour on and over-end rotation (12 rpm). The biotinylated antibody was then dialyzed (Mw cut-off value: 12.000-14.000 D) 5 times against a minimum of 50 volumes of PBS buffer (p.H 7.4) for 24 hours at 2-8°C. Turbidity was subsequently removed by sterile filtration using a 0.20 μm filter and the final volume of the biotinylated antibody was measured on a spectrophotometer at OD 280 nm, using PBS buffer at auto zero. 200 μl of biotinylated antibody solution was finally added to 800 μl buffer and measured.

### Protein extractions

Tissue taken from the injected area was pulverized in liquid nitrogen in a steel mortar. Samples were transferred into a 1.5 ml Eppendorf tube and left shaking overnight at 4°C in 0.5 M acetic acid solution containing a protease inhibitor cocktail (Roche Diagnostics, USA). The samples were then sonicated using 5 pulses at 60% amplitude (U50 control, IKA Labortechnik, Staufen, Germany), left for 2 hours gently shaking at 4°C and centrifuged for 5 minutes at 13,000 rpm. The supernatant was carefully removed, transferred to a new labelled Eppendorf tube and stored at -80°C.

### Densitometry

Densitometry measurements were performed using UN-SCAN-IT Version 6.1 from Silk Scientific (Orem, Utah 84059, USA), according to the manufacturer's guidelines. Measurements were performed for both CO3-610 and Actin loading controls related reactivity. The densitometry shown for CO3-610 was calculated relative to the loading control levels.

### Histology image analysis

Histology sections stained with Sirius red and Alcian blue were analysed using Visiopharm software Version 3.2.8.0 (Hørsholm, Denmark). The software allowed for quantification of specific tissue areas and measurement of skin thickness. Images were acquired using Pixelink PL-A623C microscope digital camera (Pixelink, Ottawa, Canada).

### SDS PAGE and Western blots

20 μg of tissue extract was mixed with loading buffer (Invitrogen LDS 4x, NP0007, California, USA), containing reducing agent (NP0004, Invitrogen). Samples were loaded into 4-12% Bis-Tris gradient gel (NP0332BOX, Invitrogen) and run for 52 minutes at 200V. Proteins were transferred onto a nitrocellulose membrane using i-Blot transfer system (Invitrogen) and blocked with 5% skimmed milk in Tris buffered Saline (TTBS) overnight at 4°C. The following antibodies were used according to the manufacturer's suggested dilutions: Beta-Actin antibody (AbCam ab8229, Cambridge, UK) was used as a loading control,

### ELISA CO3-610 Urine Assay

100 μl of Bio CO3-610 (2.5 ng/ml) in PBS-TBE was used to coat straptavidin plates for 30 minutes at 20°C on a 300 rpm shaker. Excess coater was removed by washing 5 times in washing buffer. 20 μl of each urine sample was diluted 8 fold in an incubation buffer (10 mM: 400 mM TRIS-BTB) and was then added to the plates. 100 μl of CO3-610 peroxidase conjugated antibody solution (1:40,000 dilution) was added to the plates and left for overnight incubation at 4°C, with 300 rpm shaking. Plates were washed 5 times in washing buffer. 100 μl of TMB (Kem-En-Tec A/S, Taastrup, Denmark) was added and incubated in darkness for 15 minutes at 20°C, on a 300 rpm shaker. The reaction was stopped with the addition of 100 μl of stop-solution and plates were read on an ELISA reader at 450 nm, with 650 nm as reference.

### Buffers used for ELISA

The buffer used for dissolving the coating peptide consisted of: 40 mM Na_2_HPO_4_, 12 H_2_O, 7 mM KH_2_PO_4_, 137 mM NaCl, 2.7 mM KCl, 25 mM EDTA, 0.1% Tween 20, 1% BSA, 10% sorbitol, pH 7.0. The urine assay contained 400 mM TRIZMA, 0.05% Tween 20, 0.1% BSA, 0.36% Bronidox L5, and p.H 8.0. Washing buffer was composed of 25 mM TRIZMA, 50 mM NaCl, and 0.036% Bronidox L5, 0.1% Tween 20, and the reaction-stopping buffer was composed of 0.1% H_2_SO_4_. ELISA plates used for the assay development were straptavidin-coated from Roche (Hvidovre, Denmark) cat.: 11940279. All ELISA plates were analyzed with the ELISA reader from Molecular Devices, SpectraMax M, and (CA. USA).

### Standards

The standard curve was obtained by serial dilution of biotinylated CO3-610 for the urine assay. Standard concentrations were 0, 0.33, 1, 3, 9, 27, 81 and 162 ng/ml.

### Creatinine Corrections

Creatinine levels were measured in each urine sample using the creatinine assay kit DICT-500 (BioAssay Systems Hayward, California, USA) Urine creatinine corrections were made to the CO3-610 assay results.

### Statistical analysis

Mean values and standard error of the mean (SEM) were calculated using GraphPad Prism (GraphPad Software, San Diego, CA, USA) and compared by Student's two-tailed paired t-test (α = 0.05) or by Mann-Whitney two-tailed non-parametric test, whenever appropriate. The coefficient of correlation (R^2^) and the corresponding p-value were determined by linear regression. A p-value of 0.05 was considered statistically significant. Bleomycin-treated groups were compared with PBS-treated groups for each time point of termination.

## Results

### Histology image analysis

**Figure 1:**
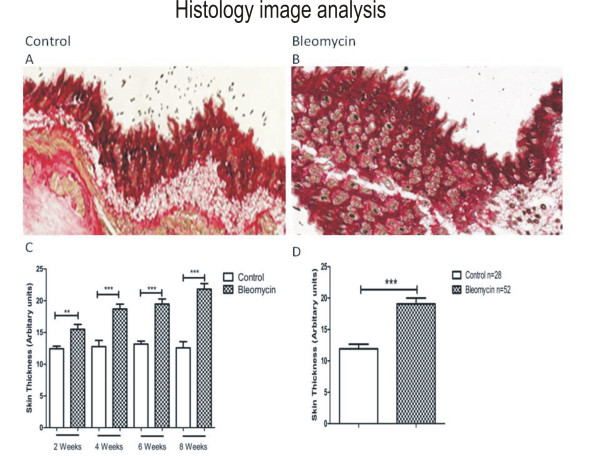
**Skin section from a control, PBS-treated mouse at 8 weeks of treatment**. (A). Skin section from a bleomycin-treated mouse at 8 weeks of treatment (B). The mean skin thickness increase between control PBS-(n = 7/time point) and bleomycin- (n = 13/time point) treated mice at 2 weeks was 12.43 vs. 15.4 arbitrary units respectively, P = 0.0029); at 4 weeks, 12.7 vs. 18.6, P = 0.0004; 6 weeks, 13.1 vs. 19.4, P < 0.0001; and at 8 weeks, 12.5 vs. 21.8, P < 0.0001 (C). Overall mean skin thickness increased between control PBS treated- (n = 28) and bleomycin- (n = 52) treated mice for the 8-week duration of the study (11.9 vs. 19.4 arbitrary units respectively, P < 0.0001). Skin thickness was calculated by Visiopharm software as an overall number per skin section, rather than by estimating density from pictures.

At the time of sacrifice, skin taken from the injected area of PBS-treated control animals showed normal gross morphology, while skin taken from the treated area of Bleomycin-injected animals showed increased collagen deposition resulting in increased skin thickness. The overall mean skin thickness in PBS- and. Bleomycin-treated animals was 11.9 and 19.4 arbitrary units respectively, representing a 63% increase (Figure 1).

### Changes in CO3-610 levels in urine

**Figure 2:**
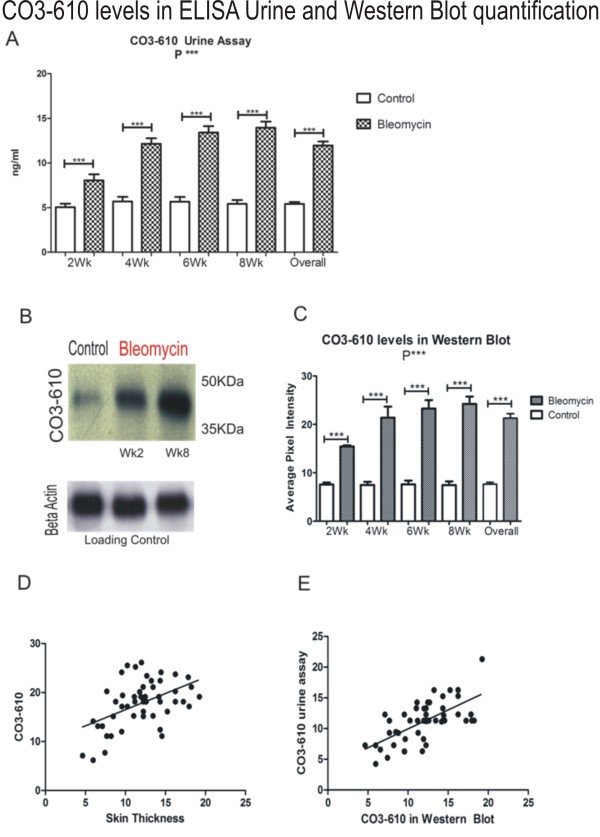
**Urine assays showed a significant increase in CO3-610 levels at all time points between bleomycin- and control PBS-treated mice**. The diagram shows the assay result at each time point (n = 7 PBS, n = 13 bleomycin-treated per termination point) and the mean CO3-610 levels across all time points (n = 28 PBS, n = 52 bleomycin-treated mice). At 2 weeks, (mean PBS 5.05 ng/ml, Bleomycin 8.04 ng/ml) P = 0.0008, 4 weeks (mean PBS 5.68 ng/ml, Bleomycin 12.1 ng/ml) P < 0.0001, 6 weeks (mean PBS 5.66 ng/ml, bleomycin 13.4 ng/ml) P < 0.0001, 8 weeks (mean PBS 5.41 ng/ml, bleomycin 13.9 ng/ml) P < 0.0001 and overall P < 0.0001 (A). CO3-610 Western blots image with control and bleomycin samples after 2 and 8 weeks treatment, and corresponding beta Actin loading controls (B). CO3-610 densitometry measurements for all time points (n = 7 PBS and n = 13 Bleomycin treated per termination point) and collective CO3-610 levels (n = 28 PBS and n = 52 bleomycin-treated mice) showing a statistically significant increase of CO3-610 levels (P < 0.0001) (C). CO3-610 levels found in the urine assay were correlated with skin thickness progression, and therefore total collagen deposition (r = 0.4883, R^2 ^= 0.2384) (D). The correlation between CO3-610 levels detected by the ELISA urine assay and Western blot densitometry measurements was statistically significant (r = 0.6528, P < 0.0001) (E).

Bleomycin-treated animals showed significantly higher levels of the MMP9-generated fragment of type III collagen, CO3-610, in urine than corresponding PBS- treated groups at the same time point. Mean values at 2 weeks were 8.044 ng/ml vs. 5.051 ng/ml, respectively), corresponding to an average increase of 59.25%. CO3-610 levels peaked after 8 weeks of bleomycin treatment. The PBS-treated group reached a peak mean CO3-610 level of 5.418 ng/ml, while the bleomycin-treated group reached a mean value 13.94 ng/ml, representing a maximum mean increase between groups of 157.29% at week 8 (Figure 2). CO3-610 levels measured by ELISA and Western blot densitometry were found to be significantly correlated (Figure 2)

## Discussion

In fibrotic lesions, the most abundant molecules in the ECM are various forms of collagens, in particular types I and III, as well as a range of proteoglycans. During fibrogenesis, levels of ECM proteins increase significantly. Thus, a marker of the turnover of these proteins may be a potential biomarker not only of skin metabolism but also turnover of internal tissue related to ECMR. Collagen type I (CO1) is the predominant collagen type and could be an attractive biomarker target. However, CO1 is degraded during bone resorption and in the fibrotic liver [18-22] and thus it is difficult to distinguish the source when measuring CO1 levels in serum or urine.

The present study demonstrates a potential alternative to CO1 as a biomarker of fibrosis. We showed that the MMP9-mediated collagen III (CO3) degradation fragment, CO3-610, can be measured in urine. Increasing CO3-610 levels were shown to be associated with skin fibrosis in mice, suggesting that CO3-610 might be a positive biomarker to study the pathogenesis of skin fibrosis in this species. We observed that CO3-610 levels increased by a mean of 157.29% compared with PBS treated animals following 8 weeks of daily bleomycin injections. CO3-610 levels as measured in the urine assay were significantly correlated to the extent of skin fibrosis. Detection limits of CO3-610 were significantly lower than those presented by Barascuk *et *al (30) in bile duct-ligated rats. IHC assessment of CO3-610 levels in skin sections could provide additional evidence of the correlation between CO3-610 and skin fibrosis. However, the main limitation of the current study was that despite our persistent attempts to use CO3-610 and a total collagen III antibody for IHC analyses on mice tissue, we were unsuccessful because we added a biotinylation step and a blocking step with mouse sera. Additional evidence of C03-610 levels in fibrotic liver sections using IHC and Western blot analyses could support the above observation.

Skin fibrosis, investigated and validated in the bleomycin model by histological staining and densitometry analysis of Western blots, was detected in the bleomycin-treated groups. The increased skin thickness due to total collagen deposition in bleomycin compared with the PBS-treated animals was found to be statistically significant at each time of termination.

## Conclusion

We provide evidence that a MMP9-cleaved collagen III degradation fragment could be a potential marker for monitoring skin fibrosis. We describe increased levels in mouse urine of the MMP-9 mediated collagen III degradation fragment CO3-610 which were correlated with skin fibrosis progression, suggesting that CO3-610 may be a potential positive biomarker to study the pathogenesis of skin fibrosis in mice. Significantly, this could have an additional clinical relevance as it could be of value for monitoring skin fibrosis and ECMR in internal organs such as the liver. Additional research in well-controlled clinical settings is needed to further investigate this finding.

## Competing interests

The research conducted was funded by Nordic Bioscience A/S

## Authors' contributions

EV: study director, study conception, immunoblotting, quantitative histology, densitometry, manuscript. SSV: study conception, manuscript, histology. NB: molecular biology, manuscript. JBM: immuno histochemistry, quantitative histology, manuscript. REC: immuno histochemistry, quantitative histology, immunoblotting manuscript. LL: molecular biology, manuscript, immunoassay. HS: immuno histochemistry, quantitative histology, manuscript. DVL: immuno histochemistry, quantitative histology, manuscript, immunoassay. ACBJ: study conception, manuscript, sample collection, statistical analysis. TSS: study conception, densitometry, manuscript, molecular biology. DJL: manuscript, immunoassay, statistical analysis. MK: study supervision, study conception, manuscript.

All authors have read and approved the final manuscript.

## Pre-publication history

The pre-publication history for this paper can be accessed here:

http://www.biomedcentral.com/1471-5945/11/6/prepub
